# Studying slippage on pushing applications with snake robots

**DOI:** 10.1186/s40638-017-0065-3

**Published:** 2017-11-02

**Authors:** Fabian Reyes, Shugen Ma

**Affiliations:** Department of Robotics, Ritsumeikan University, Kusatsu, Shiga 525-8577 Japan

**Keywords:** Snake robot, Contact, Modeling

## Abstract

In this paper, a framework for analyzing the motion resulting from the interaction between a snake robot and an object is shown. Metrics are derived to study the motion of the object and robot, showing that the addition of passive wheels to the snake robot helps to minimize slippage. However, the passive wheels do not have a significant impact on the force exerted onto the object. This puts snake robots in a similar framework as robotic arms, while considering special properties exclusive to snake robots (e.g., lack of a fixed-base, interaction with the environment through friction). It is also shown that the configuration (shape) of the snake robot, parameterized with the polar coordinates of the robot’s COM, plays an important role in the interaction with the object. Two examples, a snake robot with two joints and another with three joints, are studied to show the applicability of the model.

## Background

Robots that are capable of locomotion in unstructured conditions are necessary for realistic applications. However, locomotion alone may not be sufficient when more dexterous interaction with the environment is needed. Therefore, robotic systems with capability to locomote and also interact dexterously with their surroundings are desirable, and indeed a natural extension of robotics research.

Snake robots have shown promise regarding locomotion [1]. Locomotion in planar environments has been probably the main topic of research for snake robots [2–4] and has been extended to motion in planar slopes [5, 6], motion in 3D-space [7, 8], and more broad studies on locomotion [9]. An interesting idea that combines locomotion and interaction with the environment, called obstacle-aided locomotion (OAL), has been proposed in [10] where obstacles in the environment are used as auxiliary sources for propulsion or to avoid jamming.

**Fig. 1:**
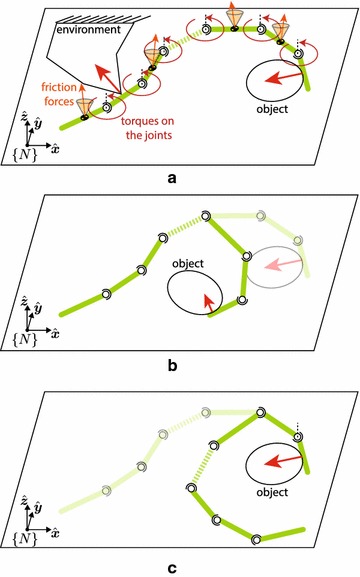
Thought experiment. General scenario of a snake robot contacting an object. **a** The snake robot contacts an object while it may also be contacting the environment either with its belly (friction) or pushing against a wall, for example. **b** The snake robot may be able to move the object. **c** The object may be very heavy and the snake robot will move around the object

Although snake robots could excel in locomotion, it is not clear if they can be used to interact with the environment (or an object) dexterously. Its structure resembles a robotic manipulator, but there are key differences that have not been fully addressed in previous research (c.f. Fig. 1).

The lack of a fixed-base makes it difficult for a snake robot to manipulate an object as dexterously as a robotic arm. Another difference is that a snake robot has contact with the environment through friction at several points of its body. Additionally, mass becomes a very important parameter to study. Unlike research regarding robotic arms where it is assumed that the arm can lift the object and it is a matter of choosing an optimal input, snake robots may not be able to move the object due to its inertial properties.

Because the kinematic structure of a snake robot resembles a robotic arm, papers that deal with similar (but not exactly the same) situations can be found in existing literature. In [11] a hyper-redundant serial robot was considered and both locomotion and manipulation of an object were considered. However, the analysis was purely kinematic while assuming a fixed-base robotic system. In other words, there was not force analysis showing the conditions for feasibility of the problem. In [12], the duality between locomotion and manipulation of a snake robot was considered under the assumption that the snake robot can be treated similarly to a robotic arm with a fixed-base when manipulating an object. This was achieved by making the first link of the snake robot behave similarly to fixed-base (due to its shape and mass), but the results cannot be extended to the case of a general snake robot. The problem of analyzing and controlling a snake robot under these conditions has been reported in [13], where it is shown that several assumptions made in previous published literature are not enough to guarantee accurate control of a planar snake robot with frictional contacts with the ground.

The main objective of this paper is to study the resulting interaction between a snake robot and an object, when the task is to push the object. We consider this to be a prelude to more interesting interactions like grasping or dexterous manipulation. However, it is important to understand the basics of the interaction first. This is an extension of previously published work [14, 15] where a more complete mathematical modeling of the problem has been presented. In [15], the optimal configurations of the snake robot to maximize the force exerted onto the object have been presented.

This paper focuses on the motion of the system, rather than forces. The main motivation for this study is that, as presented in [13], calculating an optimal input instantaneously (i.e., at one instant of time) is not enough to accurately control the system. We conjecture that understanding how the system will behave over time is also important. In other words, if the task is to push an object, then the motion of the object must be maximized, while the motion of the snake robot minimized.

Throughout this paper, there are several assumptions that have to be made because of the complexity of the problem:

1. All bodies in the system are rigid.
2. Contacts between the snake robot and objects or the environment (except the ground) are considered frictionless point contacts.
3. The snake robot has passive wheels or any other mechanical means to achieve anisotropic friction between the robot’s belly and the ground.
4. There is only one constraint per link of the snake robot.
5. The operational space is a plane (embedded in a full 3D space).
6. The snake robot has only one contact point with the object to be manipulated.

Assumption 1 allows to have a clear mathematical model of the problem without making assumptions about the compliance of the bodies which may be unrealistic to know *a priori* in real-life situations. Although this assumption may be relaxed by considering some sort of *virtual* compliance at the contacts (e.g., [16, 17]), it does not necessarily imply more realistic or correct results. In particular, it may lead to a stiff system of differential equations and several other problems. As presented in [14], a snake robot may have too many contacts with the environment leading to a statically indeterminate system [18]. In order to ensure Assumption 4, we assume that the constraints from passive wheels are removed when that link is contacting an object or a wall, for example. This can be done by lifting the links [19] or with retractable passive wheels, for example. Assumptions 1 through 4 allow to consider that all constraint forces are linearly independent and a unique solution can be found. Assumption 5 is made in order to limit the number of parameters and get clear and meaningful results which may be difficult for 3-dimensional space, since the problem presented in this paper is still very broad and has not been clearly defined as it has been discussed in this section. However, the models presented in this paper are based on spatial vectors [20] which are trivial to extend from 2D to 3D, so in the future more results can be obtained for more specific tasks. Assumption 6 is made because it is not the intent of this paper to study any type of grasp *closure* or dexterous manipulation, but to understand the interaction itself first.

The paper is organized as follows. In “Mathematical background” section, the necessary mathematical background to understand this paper is presented along references necessary to develop the concepts further. “Motion of the system due to the interaction” section is the main body of the paper; the modeling of the system is presented, and metrics and quantities mentioned in this section are derived. In “Results” section, a specific example is studied to show the application of the proposed metrics. “Discussion and future applications” section includes several comments regarding the scope and limitations of the results presented in this paper. The paper concludes with some remarks in “Conclusion” section.

## Mathematical background

In this section, we give a very brief introduction to the mathematical topics necessary to understand this paper. We recommend [20–22] for a more detailed treatment. In particular, the foundations of the model used in this paper have been presented in [15]; readers are encouraged to read this reference for a more detailed treatment of snake robots in the framework of articulated-bodies. As stressed in previous research, it is important to guarantee invariance of metrics in order for the results to be meaningful. Not only to avoid inconsistency of units, but also for the metric to be invariant to a change of coordinates. To derivate the analysis of the system to lead to meaningful metrics, we employ dual vectors [23] and basic differential geometry [21].

### Differential geometry: twists, wrenches, and metrics

A twist (concatenation of linear and angular velocity) ${\vec { \varvec{\upsilon }} \in {\text{M}}^{n}}$ can be expressed w.r.t. a covariant basis ${\varvec{e} = [\vec {\varvec{e}}_{1}, \ldots , \vec {\varvec{e}}_{n}]^{\mathrm{T}}}$ as ${\vec { \varvec{\upsilon }} = \varvec{e}^{\mathrm{T}} \varvec{\upsilon }}$. The element $\varvec{\upsilon }\in {\mathfrak{R}}^{n}$ can be interpreted as the (vector of) contravariant components of $\vec {\varvec{\upsilon }}$. A wrench (concatenation of linear force and torque) ${\vec { \varvec{f}} \in \text{ F }^{n}}$ can be expressed w.r.t. a contravariant basis ${\varvec{e}^{*} = [\vec {\varvec{e}}_{1}^{*},\ldots ,\vec {\varvec{e}}^{*}_{n}]^{\mathrm{T}}}$ as ${\vec {\varvec{f}} = \varvec{e}^{*^{\mathrm{T}}} \varvec{f}^{*}}$ and $\varvec{f}^{*} \in {\mathfrak{R}}^{n}$ can be interpreted as the covariant components of $\vec {\varvec{f}}$.

It is important to notice that both bases $\varvec{e}$ and $\varvec{e}^{*}$ may not be orthogonal, so the common definition of inner product (e.g., $\varvec{\upsilon } \cdot \varvec{\upsilon } = \varvec{\upsilon }^{\mathrm{T}} \varvec{\upsilon }$) would give incorrect results. Let us denote the metric tensor of the covariant basis as $\varvec{I} = \varvec{e} \varvec{e}^{\mathrm{T}}$ and its inverse by $\varvec{I}^{-1}$. The (squared) length of a twist and wrench is an invariant quantity and can be obtained using the scalar product $\{\circ \}$ while taking into account the metric tensor as

1 $$\begin{aligned} ||\vec {\varvec{\upsilon }}||^{2}=  \vec {\varvec{\upsilon }} \circ \vec {\varvec{\upsilon }} = \varvec{\upsilon }^{\mathrm{T}} \varvec{e} \varvec{e}^{\mathrm{T}} \varvec{\upsilon } = \varvec{\upsilon }^{\mathrm{T}} \varvec{I} \varvec{\upsilon }, \end{aligned}$$

2 $$\begin{aligned} ||\vec {\varvec{f}}||^{2}=  \vec {\varvec{f}} \circ \vec {\varvec{f}} = \varvec{f}^{*^T} \varvec{e}^{*} \varvec{e}^{*^T} \varvec{f} = \varvec{f}^{\mathrm{T}} \varvec{I}^{-1} \varvec{f}. \end{aligned}$$

### Unconstrained model of the snake robot

**Fig. 2:**
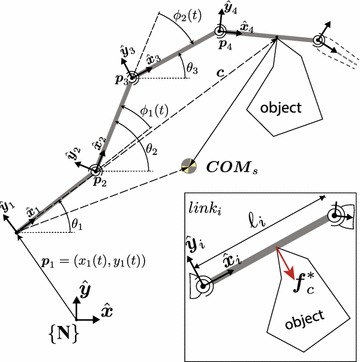
Kinematic model. The generalized coordinates of the snake robot and its COM are shown. Also, the interaction between the snake robot and object with its corresponding contact force can be seen $\varvec{f}_{c}^{*}$

The snake robot can be modeled as a series of rigid links connected by revolute joints. All joints have their axes parallel to each other; therefore, the snake robot is constrained to move on a plane (but is unconstrained in any other way). The kinematic model of a snake robot is similar to an open-chain robotic manipulator (c.f. Fig. 2). The model has been previously studied in [13, 15, 24, 25].

The snake robot has a total of ${n_{s} \in \mathbb {N}}$ degrees of freedom (DOFs), and its generalized coordinates are encapsulated in the vector $\varvec{q}_{s}(t) \in {\mathfrak{R}}^{n_{s}}$. The snake robot has $n_{\ell }=n_{a}+1$ links each with mass $m_{i}$.

The Jacobian for the *i*th link is a mapping from the vector of generalized velocities $\dot{\varvec{q}}_{s}$ to the twist $\varvec{\upsilon }_{i} \in {\mathfrak{R}}^{3}$ of the link and is denoted as $\varvec{J}_{i} \in {\mathfrak{R}}^{3 \times n_{s}}$

3 $$\begin{aligned} \varvec{\upsilon }_{i} = \varvec{J}_{i} \dot{\varvec{q}}_{s}. \end{aligned}$$

The equations of motion of the snake robot can be presented in the canonical form

4 $$\begin{aligned} \varvec{e}^{*^{\mathrm{T}}} \left[ \varvec{M}_{s} \ddot{\varvec{q}}_{s} + \varvec{h}_{s}^{*} \right] = \varvec{e}^{*^{\mathrm{T}}} \left[ \varvec{B} \varvec{\tau }_{\mathrm{act}}^{*} + \varvec{\tau }_{\mathrm{ext}}^{*} \right] \end{aligned}$$

where ${\varvec{M}_{s} (\varvec{q}_{s}) \in {\mathfrak{R}}^{n_{s} \times n_{s}}}$ is the inertia matrix of the snake robot (a symmetric positive definite (PD) matrix), $\varvec{h}_{s}^{*} (\varvec{q}_{s}, \dot{\varvec{q}}_{s})\in {\mathfrak{R}}^{n_{s} \times 1}$ contains Coriolis and centripetal effects, and $\varvec{\tau }_{\mathrm{ext}}^{*} (\varvec{q}, \dot{\varvec{q}}) \in {\mathfrak{R}}^{n_{s} \times 1}$ is a vector of torques produced by external forces (e.g., kinetic friction). The matrix ${\varvec{B} \in {\mathfrak{R}}^{n_{s} \times n_{a}}}$ defined as

5 $$\begin{aligned} \varvec{B} := \left[ \begin{array}{c} \varvec{0}_{3 \times n_{a}}\\ \varvec{1}_{n_{a} \times n_{a}} \end{array}\right] , \end{aligned}$$

is a matrix that projects the vector of input forces $\varvec{\tau }_{\mathrm{act}}^{*}$ into the space of generalized forces. The matrix $\varvec{1}$ denotes the identity matrix of appropriate dimensions.

### Unconstrained model of an object

A rigid body is able to move in its operational space with dimensions $n_{\mathrm{op}},$ and its equations of motion can be compactly written as

6 $${\varvec{e}}^{*{\text{T}}} \left[{\varvec{I}}_{\text{obj}} {\varvec{a}}_{\text{obj}} + {\varvec{p}}_{\text{obj}}^{*} \right] = {\varvec{e}}^{*{\text{T}}} \left[{\varvec{f}}_{\text{obj}}^{*} \right],$$

where ${\varvec{a}_{\mathrm{obj}}}$, ${\varvec{p}_{\mathrm{obj}}^{*}}$, and ${\varvec{f}_{\mathrm{obj}}^{*} \in {\mathfrak{R}}^{n_{\mathrm{op}}}}$ denote the acceleration, velocity-produced terms, and total wrench acting on the body, respectively. If the body is constrained to move in a plane (but unconstrained in any other way), it will have three DOFs (i.e., $n_{\mathrm{op}} = 3$). ${\varvec{I}_{\mathrm{obj}} \in \text{ R }^{n_{\mathrm{op}} \times n_{\mathrm{op}}}}$ denotes the inertia tensor of the rigid body. The mass of the object $m_{\mathrm{obj}}$ will be denoted as a multiple of the mass of a link of the snake robot as $m_{\mathrm{obj}} = \kappa m_{i}$. In other words, $\kappa$ is a proportionality coefficient relating the masses of interest.

If all links of the snake robot have the same mass *m*, then the inertia matrix of the snake robot can be factored as $\varvec{M}_{s} := m \bar{\varvec{M}}$, and the inertia matrix of an object as $\varvec{I}_{\mathrm{obj}} = m_{\mathrm{obj}} \bar{\varvec{I}}_{\mathrm{obj}}$, where the new inertia matrices $\bar{\varvec{M}}$ and $\bar{\varvec{I}}_{\mathrm{obj}}$ correspond to inertia matrices with unitary mass.

### Summary of constraints

The interaction between a snake robot and an external object creates a set of forces between them that avoid penetration (also called kinematic constraints or non-penetrability constraints [21, 26, 27]). Additionally, the (static) friction forces between the belly of the robot and the ground can also be modeled as constraint forces (bounded by their friction limit). Assuming there are $n_{c} \in \mathbb {N}$ constraint forces in total, the constraint forces ${\varvec{f}_{c}^{*} \in {\mathfrak{R}}^{n_{c}} }$ span the constrained subspace

7 $$\begin{aligned} \mathcal {C} = \lbrace \varvec{f}_{c}^{*} : \varvec{f}_{c}^{*} = \varvec{T} \varvec{\lambda }^{*} \rbrace , \end{aligned}$$

where the matrix $\varvec{T} \in ^{n_{\mathrm{op}}n_{c} \times n_{c}}$ is a matrix spanning the constraint forces on the operational space [21], and $\varvec{\lambda }^{*} \in {\mathfrak{R}}^{n_{c}}$ contains the magnitude of the constraint forces (in the context of optimization this vector is usually called the Lagrangian multipliers [21, 26]).

To facilitate the coupling between the snake robot and environment/object(s), it is useful to put together all the constraints in vector/matrix form. All the constraints can be put together into the following form

8 $$\begin{aligned} \varvec{A} \left[ \begin{array}{c} \dot{\varvec{q}}_{s} \\ \varvec{\upsilon }_{\mathrm{obj}} \end{array}\right] \geqslant \varvec{0} \end{aligned}$$

where the $\varvec{A} \in {\mathfrak{R}}^{n_{s} \times n}$ is called the constraint matrix and takes the following form

9 $$\begin{aligned} \varvec{A} = \left[ \begin{array}{ll} -\varvec{J}_{s}&\varvec{G}^{\mathrm{T}} \end{array}\right] , \end{aligned}$$

where $\varvec{J}_{s} \in {\mathfrak{R}}^{n_{c} \times n_{s}}$ is called the robot Jacobian (also called *hand Jacobian* [26, 27]) which projects the vector of generalized velocities of the snake robot onto the constrained subspace

10 $$\begin{aligned} \varvec{J}_{s} = \left[ \begin{array}{ccc} \varvec{T}_{1}^{\mathrm{T}} &{} \cdots &{} \varvec{0}\\ \varvec{0} &{} \ddots &{} \varvec{0}\\ \varvec{0} &{} \cdots &{} \varvec{T}_{n_{c}}^{\mathrm{T}} \end{array}\right] \left[ \begin{array}{c} \varvec{J}_{*}\\ \vdots \\ \varvec{J}_{*} \end{array}\right] , \end{aligned}$$

where $\varvec{T}_{k}$ spans the constrained space for the *k*th constraint and $\varvec{J}_{*}$ denotes the Jacobian corresponding to the link under that constraint, without any specific ordering. The matrix ${\varvec{G} \in {\mathfrak{R}}^{n_{\mathrm{op}} \times n_{c}}}$ is usually referred to as *Grasp Matrix* and its transpose is a mapping from the motion space of the object to the constrained subspace; it can be constructed in a similar manner to the robot Jacobian. The constraint forces projected back onto the snake robot and object are

11 $$\begin{aligned} \left[ \begin{array}{c} \varvec{\tau }_{c}^{*} \\ \varvec{f}_{c}^{*} \end{array}\right] = \left[ \begin{array}{c}\varvec{-} \varvec{J}_{s}^{\mathrm{T}} \\ \varvec{G}\end{array}\right] \varvec{\lambda }^{*} = \varvec{A}^{\mathrm{T}} \varvec{\lambda }^{*}, \end{aligned}$$

where $\varvec{\tau }_{c}^{*} \in {\mathfrak{R}}^{n_{s}}$ is simply the projection of the constraint reaction force $- \varvec{f}_{c}^{*}$ onto the space of generalized forces of the snake robot.

## Motion of the system due to the interaction

Now that it is assumed that the snake robot is touching at least one object, the new coupled equations of motion can be written as

12 $$\begin{aligned} \varvec{e}^{*T} \left[ \varvec{I} \varvec{a} + \varvec{p}^{*} \right]= \varvec{e}^{*T} \left[ \varvec{f}^{*} + \varvec{A}^{\mathrm{T}} \varvec{\lambda }^{*} \right] ,\end{aligned}$$

$$\begin{aligned} \varvec{I}&= \left[ \begin{array}{ll} \varvec{M}_{s} &{} \varvec{0} \\ \varvec{0} &{} \varvec{I}_{\mathrm{obj}} \end{array}\right] \qquad \varvec{a} = \left[ \begin{array}{l} \ddot{\varvec{q}}_{s} \\ \varvec{a}_{\mathrm{obj}} \end{array}\right]  \\ \varvec{p}^{*}&= \left[ \begin{array}{l} \varvec{h}_{s}^{*} \\ \varvec{p}_{\mathrm{obj}}^{*} \end{array}\right] \qquad \varvec{f}^{*} = \left[ \begin{array}{l} \varvec{B} \varvec{\tau }_{\mathrm{act}}^{*} \\ \varvec{f}_{\mathrm{obj}}^{*} \end{array}\right] \end{aligned}$$

along the constraints

13 $$\begin{aligned} \varvec{A} \varvec{a} + \dot{\varvec{A}} \varvec{\upsilon }\geqslant \varvec{0}, \end{aligned}$$

where equality holds for constraints imposed by friction. Equation (13) is the derivative of (8). As discussed in [21], both holonomic and non-holonomic constraints can be obtained in this uniform manner at the acceleration level.

The change from the contravariant basis $\varvec{e}^{*}$ to the basis of the constrained space $\varvec{e}_{c}^{*}$ can be obtained by using the projector ${{}^{\varvec{e}_{c}^{*}} \varvec{\Phi }_{\varvec{e}^{*}}: {\mathfrak{R}}^{n} \rightarrow {\mathfrak{R}}^{n_{c}}}$ [15, 21] defined as

14 $$\begin{aligned} {}^{\varvec{e}_{c}^{*}} \varvec{\Phi }_{\varvec{e}^{*}} := (\varvec{A} \varvec{I}^{-1} \varvec{A}^{\mathrm{T}})^{-1} \varvec{A} \varvec{I}^{-1} = \varvec{G}_{c}^{-1} \varvec{A} \varvec{I}^{-1}. \end{aligned}$$

The positive semidefinite (PSD) matrix ${\varvec{G}_{c}:=(\varvec{A} \varvec{I}^{-1} \varvec{A}^{\mathrm{T}})}$ represents the metric tensor for the basis $\varvec{e}_{c}^{*}$ and has full rank if all constraints are linearly independent. (Additional comments regarding the rank of this metric tensor are located in “Discussion and future applications” section). The mapping (14) can be interpreted as the left pseudo inverse of the matrix $\varvec{A}^{\mathrm{T}}$ as

15 $$\begin{aligned} \varvec{A}^{T^{\dagger }} :=(\varvec{A} \varvec{I}^{-1} \varvec{A}^{\mathrm{T}})^{-1} \varvec{A} \varvec{I}^{-1} \rightarrow \varvec{A}^{T^{\dagger }} \varvec{A}^{\mathrm{T}} = \varvec{1}. \end{aligned}$$

The constraint forces $\vec {\varvec{\lambda }}=\varvec{e}_{c}^{*T} \varvec{\lambda }^{*}$ can be obtained by projecting the equations of motion (12) onto the constrained subspaces using the projector (14) and taking into account the constraint (13) as

16 $$\begin{aligned} \varvec{e}_{c}^{*T} \varvec{\lambda }^{*} \geqslant \varvec{e}_{c}^{*T} \left[ - \varvec{A}^{T^{\dagger }} \varvec{f}^{*} + (\varvec{A} \varvec{I}^{-1} \varvec{A}^{\mathrm{T}})^{-1} (\varvec{A} \varvec{I}^{-1} \varvec{p}^{*} - \dot{\varvec{A}} \varvec{\upsilon }) \right] . \end{aligned}$$

The right-hand side (RHS) of (16) has two terms. The first term depends purely on the set of forces exerted onto the system, either by the actuators of the snake robot or an external wrench exerted onto the object. The second term includes terms produced by velocity and will vanish if the system starts from an equilibrium configuration. This (affine) system of equations is usually interpreted as a force ellipsoid [27–29], and it maps a quadratic region in the input space $\varvec{f}^{*}$ to an ellipsoid in the output space of constraint forces $\varvec{\lambda }^{*}$, while the velocity-produced terms will shift the origin of such ellipsoid. In this paper, it is assumed the system starts from equilibrium so that the following linear mapping can be defined

17 $$\begin{aligned} \varvec{\lambda }^{*} = - {}^{\varvec{e}_{c}^{*}} \varvec{\Phi }_{\varvec{e}^{*}} \varvec{f}^{*}. \end{aligned}$$

The obtained contrained forces (due to the inputs in the system) can be substituted back onto the equations of motion (12). The resulting acceleration of the system is

18 $$\begin{aligned} \varvec{e}^{\mathrm{T}} \varvec{a} = \varvec{e}^{T} \left[ \varvec{I}^{-1} ({}^{\varvec{e}_{c}^{*\perp }} \varvec{\Phi }_{\varvec{e}^{*}}) \varvec{f}^{*} \right] , \end{aligned}$$

where the new projector ${}^{\varvec{e}_{c}^{*\perp }} \varvec{\Phi }_{\varvec{e}^{*}}: {\mathfrak{R}}^{n} \rightarrow {\mathfrak{R}}^{n}$ defined as

19 $$\begin{aligned} {}^{\varvec{e}_{c}^{*\perp }} \varvec{\Phi }_{\varvec{e}^{*}} := \varvec{1} - \varvec{A}^{\mathrm{T}} \varvec{A}^{T^{\dagger }} \end{aligned}$$

is a projector from input wrenches $\varvec{e}^{*T} \varvec{f}^{*}$ to the space orthogonal to the constrained space $\mathcal {C}$, but with coordinates expressed with respect to the original basis $\varvec{e}^{*}$. The extra inverse inertia $\varvec{I}^{-1}$ transforms to coordinates in $\varvec{e}$ basis (i.e., transforms from wrenches to twists).

By solving for $\ddot{\varvec{q}}_{s}$ and $\varvec{a}_{\mathrm{obj}}$, the motion of the snake robot and object can be obtained as

20 $$\begin{aligned} \ddot{\varvec{q}}_{s}= \left( \varvec{}^{\ddot{\varvec{q}}_{s}} \varvec{\Phi }_{\varvec{\tau }_{\mathrm{act}}^{*}} \right) \varvec{\tau }_{\mathrm{act}}^{*} + \left( \varvec{}^{\ddot{\varvec{q}}_{s}} \varvec{\Phi }_{\varvec{f}_{\mathrm{obj}}^{*}} \right) \varvec{f}_{\mathrm{obj}}^{*},\end{aligned}$$

21 $$\begin{aligned} \varvec{a}_{\mathrm{obj}}= \left( \varvec{}^{\varvec{a}_{\mathrm{obj}}} \varvec{\Phi }_{\varvec{\tau }_{\mathrm{act}}^{*}} \right) \varvec{\tau }_{\mathrm{act}}^{*} + \left( \varvec{}^{\varvec{a}_{\mathrm{obj}}} \varvec{\Phi }_{\varvec{f}_{\mathrm{obj}}^{*}} \right) \varvec{f}_{\mathrm{obj}}^{*}, \end{aligned}$$

where the auxiliary mappings ${\varvec{}^{\ddot{\varvec{q}}_{s}} \varvec{\Phi }_{\varvec{\tau }_{\mathrm{act}}^{*}}: {\mathfrak{R}}^{n_{a}} \rightarrow {\mathfrak{R}}^{n_{s}}}$, ${\varvec{}^{\ddot{\varvec{q}}_{s}} \varvec{\Phi }_{\varvec{f}_{\mathrm{obj}}^{*}}: {\mathfrak{R}}^{n_{\mathrm{op}}} \rightarrow {\mathfrak{R}}^{n_{s}}}$,

${\varvec{}^{\varvec{a}_{\mathrm{obj}}} \varvec{\Phi }_{\varvec{\tau }_{\mathrm{act}}^{*}}: {\mathfrak{R}}^{n_{a}} \rightarrow {\mathfrak{R}}^{n_{\mathrm{op}}}}$, and ${\varvec{}^{\varvec{a}_{\mathrm{obj}}} \varvec{\Phi }_{\varvec{f}_{\mathrm{obj}}^{*}} : {\mathfrak{R}}^{n_{\mathrm{op}}} \rightarrow {\mathfrak{R}}^{n_{\mathrm{op}}}}$ can be defined as

22 $$\begin{aligned} \varvec{}^{\ddot{\varvec{q}}_{s}} \varvec{\Phi }_{\varvec{\tau }_{\mathrm{act}}^{*}}:=  \frac{1}{m} \bar{\varvec{M}}_{s}^{-1} \left( \varvec{1} - \varvec{J}_{s}^{\mathrm{T}} \left( \varvec{G}_{c}^{-1} \right) \varvec{J}_{s} \bar{\varvec{M}}_{s}^{-1} \right) \varvec{B} \end{aligned}$$

23 $$\begin{aligned} \varvec{}^{\ddot{\varvec{q}}_{s}} \varvec{\Phi }_{\varvec{f}_{\mathrm{obj}}^{*}}:= \frac{1}{\kappa m} \bar{\varvec{M}}_{s}^{-1} \varvec{J}_{s}^{\mathrm{T}} \left( \varvec{G}_{c}^{-1} \right) \varvec{G}^{\mathrm{T}} \bar{\varvec{I}}_{\mathrm{obj}}^{-1} \end{aligned}$$

24 $$\begin{aligned} \varvec{}^{\varvec{a}_{\mathrm{obj}}} \varvec{\Phi }_{\varvec{\tau }_{\mathrm{act}}^{*}}:= \frac{1}{\kappa m} \bar{\varvec{I}}_{\mathrm{obj}}^{-1} \varvec{G} \left( \varvec{G}_{c}^{-1} \right) \varvec{J}_{s} \bar{\varvec{M}}_{s}^{-1} \varvec{B} \end{aligned}$$

25 $$\begin{aligned} \varvec{}^{\varvec{a}_{\mathrm{obj}}} \varvec{\Phi }_{\varvec{f}_{\mathrm{obj}}^{*}}:= \frac{1}{\kappa m} \bar{\varvec{I}}_{\mathrm{obj}}^{-1} \left( \varvec{1}-\frac{1}{\kappa } \varvec{G} \left( \varvec{G}_{c}^{-1} \right) \varvec{G}^{\mathrm{T}} \bar{\varvec{I}}_{\mathrm{obj}}^{-1} \right) \end{aligned}$$

The (squared) length of the accelerations of the snake robot $||\vec {\ddot{\varvec{q}}}_{s}||^{2}$ or object $||\vec {\varvec{a}}_{\mathrm{obj}}||^{2}$ can be obtained in an invariant way by considering its metric tensors, where the total expression can be divided into three terms as

26 $$\begin{aligned} ||\vec {\ddot{\varvec{q}}}_{s}||^{2}= \varvec{\tau }_{\mathrm{act}}^{*T} \left( \varvec{\Xi }_{\varvec{\tau }_{\mathrm{act}}^{*}} \right) \varvec{\tau }_{\mathrm{act}}^{*} + \varvec{f}_{\mathrm{obj}}^{*T} \left( \varvec{\Xi }_{\varvec{f}_{\mathrm{obj}}^{*}} \right) \varvec{f}_{\mathrm{obj}}^{*} + \varvec{\tau }_{\mathrm{act}}^{*T} \left( {}_{\varvec{\tau }_{\mathrm{act}}^{*}} \varvec{\Xi }_{\varvec{f}_{\mathrm{obj}}^{*}} \right) \varvec{f}_{\mathrm{obj}}^{*} \end{aligned}$$

27 $$\begin{aligned} ||\vec {\varvec{a}}_{\mathrm{obj}}||^{2}= \varvec{\tau }_{\mathrm{act}}^{*T} \left( \varvec{\Omega }_{\varvec{\tau }_{\mathrm{act}}^{*}} \right) \varvec{\tau }_{\mathrm{act}}^{*} + \varvec{f}_{\mathrm{obj}}^{*T} \left( \varvec{\Omega }_{\varvec{f}_{\mathrm{obj}}^{*}} \right) \varvec{f}_{\mathrm{obj}}^{*} + \varvec{\tau }_{\mathrm{act}}^{*T} \left( {}_{\varvec{\tau }_{\mathrm{act}}^{*}} \varvec{\Omega }_{\varvec{f}_{\mathrm{obj}}^{*}} \right) \varvec{f}_{\mathrm{obj}}^{*}. \end{aligned}$$

We will concentrate on the contributions of the inputs of the snake robot. It can be verified that the auxiliary mappings $\varvec{\Xi }_{\varvec{\tau }_{\mathrm{act}}^{*}}$ and $\varvec{\Omega }_{\varvec{\tau }_{\mathrm{act}}^{*}}$, after some manipulation, can be defined as

28 $$\begin{aligned} \varvec{\Xi }_{\varvec{\tau }_{\mathrm{act}}^{*}}:=  \frac{1}{m} \varvec{B}^{\mathrm{T}} \left( \varvec{1}- \hat{\varvec{J}}_{s}^{\mathrm{T}} \right) ^{\mathrm{T}} \varvec{M}_{s}^{-1} \left( \varvec{1}- \hat{\varvec{J}}_{s}^{\mathrm{T}} \right) \varvec{B} \end{aligned}$$

29 $$\begin{aligned} \varvec{\Omega }_{\varvec{\tau }_{\mathrm{act}}^{*}}:=  \frac{1}{\kappa m} \varvec{B}^{\mathrm{T}} \bar{\varvec{M}}_{s}^{-1} \varvec{J}_{s}^{\mathrm{T}} \varvec{G}_{c}^{-1} \varvec{G}^{\mathrm{T}} \bar{\varvec{I}}_{\mathrm{obj}}^{-1} \varvec{G} \varvec{G}_{c}^{-1} \varvec{J}_{s} \bar{\varvec{M}}_{s}^{-1} \varvec{B} \end{aligned}$$

where the auxiliary term

$$\begin{aligned} \hat{\varvec{J}}_{s}^{\mathrm{T}} := \varvec{J}_{s}^{\mathrm{T}} \varvec{G}_{c}^{-1} \varvec{J}_{s} \bar{\varvec{M}}_{s}^{-1} \end{aligned}$$

has been introduced for a more compact notation and any further simplification has been omitted for simplicity’s sake. However, the linear relationship w.r.t. the masses of the system becomes evident.

### Slippage ratio

As stated in “Background” section, it is an important problem to predict motion and not only forces, in order to understand the interaction between the snake robot and object and try to accomplish a task. If the task is to manipulate an object, then it is desirable to maximize the motion of the object $||\vec {\varvec{a}}_{\mathrm{obj}}||^{2}$ while minimizing the slippage of the snake robot $||\vec {\ddot{\varvec{q}}}_{s}||^{2}$. On the other hand, a snake robot could locomote using the environment as a source of propulsive forces or as a support, similar to the idea of climbing [30–32]. This case resembles more a walking robot where the contact with the environment is necessary for the robot to move. To the best of our knowledge, this distinction has not been studied with snake robots. To analyze this, we propose the ratio of accelerations

30 $$\begin{aligned} sr := \frac{||\vec {\varvec{a}}_{\mathrm{obj}}||^{2}}{||\vec {\varvec{a}}_{\mathrm{obj}}||^{2}+||\vec {\ddot{\varvec{q}}}_{s}||^{2}}, \end{aligned}$$

and call it *slippage ratio* which is a dimensionless scalar quantity bounded as ${sr \in [0,1]}$. Using this ratio, we can analyze the following three general situations:

- $\hbox {sr} \rightarrow 1$ which implies that the acceleration of the snake robot is minimal (${||\vec {\ddot{\varvec{q}}}_{s}||^{2} \ll ||\vec {\varvec{a}}_{\mathrm{obj}}||^{2}}$ or ${||\vec {\ddot{\varvec{q}}}_{s}||^{2} \approx 0}$).
- $\hbox {sr} \approx 0.5$ which implies a similar magnitude of acceleration for the two subsystems (${||\vec {\ddot{\varvec{q}}}_{s}||^{2} \approx ||\vec {\varvec{a}}_{\mathrm{obj}}||^{2}}$).
- $\hbox {sr} \rightarrow 0$ which implies that the magnitude of the acceleration of the object is minimal (${||\vec {\ddot{\varvec{q}}}_{s}||^{2} \gg ||\vec {\varvec{a}}_{\mathrm{obj}}||^{2}}$ or ${||\vec {\varvec{a}}_{\mathrm{obj}}||^{2} \approx 0}$).

This quantity can be seen as the ratio between a *desired* output and the total output. By analyzing the slippage ratio sr, given a configuration and input, we can understand better the behavior of the system.

### Polar coordinates of the COM of the snake robot

**Fig. 3:**
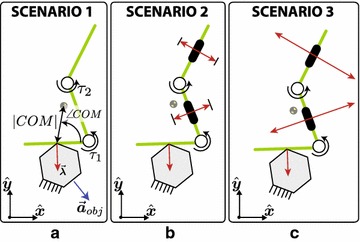
Three scenarios. Snake robot with 2 joints contacting an object. **a** The friction between the snake robot and the ground is negligible. **b** The snake robot has passive wheels (represented by the black rectangles) with a limit surface for the friction force. **c** Ideal passive wheels are assumed

In order to compare snake robots with different number of joints, it is necessary to parameterize the configuration of the robot with a set of parameters in common. The idea of using the polar coordinates of the COM of the snake robot w.r.t. the contact point with the object has been introduced in [15] and further explored in [24]. The (unsigned) distance from the COM of the snake robot to the contact point is denoted by |*COM*|, and the angle between this vector and the link contacting the object is denoted as $\angle {COM}$. These quantities can be seen in Fig. 3.

## Results

To study the interaction between snake robot and object, we can apply the framework proposed in this paper while changing the number and type of constraints and studying the resulting accelerations of the system. In general, we propose three different scenarios depending on the type of constraints present on the system as follows:

- Scenario 1: The snake robot is in contact with an object but unconstrained in any other way. The friction between the snake robot and ground is negligible.
- Scenario 2: The snake robot contacts one object and has passive wheels in all other links. The friction between the passive wheels and ground is bounded by its limit surface.
- Scenario 3: The snake robot is contacting one object and has passive wheels in all other links. The passive wheels impose (unbounded and bilateral) non-holonomic constraints.

In other words, we will change the properties of the interaction between the snake robot and the environment (ground) and then analyze the resulting acceleration of the object $||\vec {\varvec{a}}_{\mathrm{obj}}||^{2}$, of the snake robot $||\vec {\ddot{\varvec{q}}}_{s}||^{2}$ and the slippage ratio sr as a result. Scenario 1 allows us to consider only the inertial properties of the system. Scenario 2, on the other hand, allows us to study the effect that passive wheels have on the system, but with a bounded friction coefficient $\mu _{s}$. Scenario 3 considers ideal passive wheels and could be considered as the extreme case when $\mu _{s} \rightarrow \infty$. This is the most common model used for studying locomotion of snake robots.

The norms (26), (27) and slippage ratio (30) have to be studied over regions of the input space on a specific *k*th configuration. The inputs are restricted to the quadratic region of the input

31 $$\begin{aligned} \bar{\tau } = \{ \varvec{\tau }_{\mathrm{act}}^{*}: \varvec{\tau }_{\mathrm{act}}^{*T} \left( \bar{\varvec{M}}_{s}^{-1}\right) \varvec{\tau }_{\mathrm{act}}^{*} \leqslant 1 \} \end{aligned}$$

which will be, in general, an ellipsoid and not a unitary sphere as is usually considered (i.e., $||\vec {\varvec{\tau }}_{\mathrm{act}}||^{2} \leqslant 1$ is not the same as $\varvec{\tau }_{\mathrm{act}}^{*T} \varvec{\tau }_{\mathrm{act}}^{*} \leqslant 1$).

### Case study 1: Snake robot with two joints

In order to show more specific and qualitative results, we apply the mappings and study a snake robot with two joints (c.f. Fig. 3). The small number of joints allows us to show graphically the magnitude of the studied norms as a function of the joint torques. First, we study a snake robot with the parameters described in Table 1. The snake robot is contacting an object with its tail (first link) and the contact occurs at the middle of the link (c.f. Fig. 3). The angles of the joints are varied in the range [− 135°, 135°] every 10° (784 configurations in total), and the metrics (26), (27), and (30) are calculated within the quadratic region (31).

**Table 1:** Parameters of the simulation for case study 1

Symbol	Value	Unit	Description
*n*	5		Number of DOFs of the system
$n_{a}$	2		Number of actuated joints
$$ {m}_{i}$$	1	(kg)	Mass of $$link_{i}$$, $$i=1,\ldots ,n_{\ell }$$
$\ell _{i}$	0.15	(m)	Length of $link_{i}$, $1=1,\ldots ,n_{\ell }$
$I_{{\mathrm{com}},i}$	0.002	(kg m^2^)	Rotational inertia for the *i*th link
$\varvec{\tau }_{\mathrm{act}} = [\tau _{a1}, \tau _{a2}]^{\mathrm{T}}$		(N m)	Input joint torques
$\mu _{s}$	0.1		Coefficient of (static) friction used for Scenario 2

**Fig. 4:**
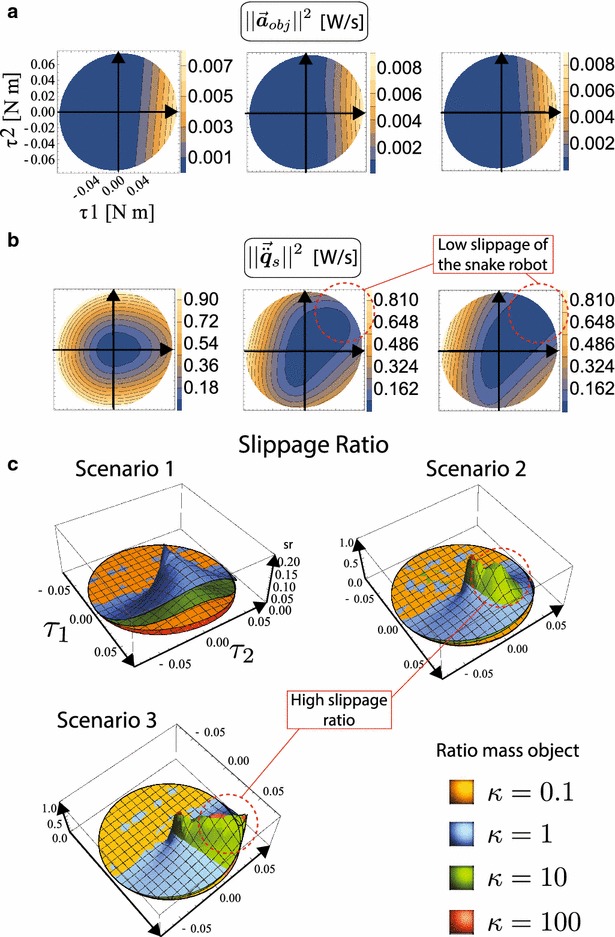
Norms of motion of the system. The first, second, and third column represent scenario 1, scenario 2, and scenario 3, respectively. A higher value represents more power transmitted to the respective motion. The configuration of the robot is $\varvec{q}_{s} = \{ 0,0,0,-135^{\circ },-\,135^{\circ }\}$. **a** Acceleration of the object. **b** Acceleration of the snake robot. **c** Slippage ratio. Several values of $\kappa$ are shown

One example configuration can be seen in Fig. 4, where it is assumed that the object has a hundred times the mass of a link of the robot (i.e., $\kappa =100$). The first, second, and third columns represent the three scenarios depicted in Fig. 3, respectively. A lighter color represents a higher value of the depicted norm. Figure 4a shows the magnitude of the acceleration of the object $||\vec {\varvec{a}}_{\mathrm{obj}}||^{2}$. It can be seen that it barely changes regardless of the scenario (i.e., independently of the fact that the snake robot has or has not passive wheels, the object will accelerate the same given the same input). Figure 4b shows the magnitude of the acceleration of snake robot $||\vec {\ddot{\varvec{q}}}_{s}||^{2}$. This shows clearly that, even if the object’s acceleration is similar for all three scenarios, the behavior of the snake robot changes. The addition of passive wheels (second and third columns) increases the area where the snake robot’s slippage is minimal. Without passive wheels, the snake robot will slip in almost any direction of the input space.

The slippage ratio (30) gives quantitative information about the movement of the system and can be studied in the same way as the previous norms. Figure 4c shows the value of the slippage ratio for all three scenarios with several values for $\kappa$ for one configuration. It can be seen that $\hbox {sr} \rightarrow 0$ in the region where there is no contact with the object (i.e., the snake robot can move freely and therefore $||\vec {\varvec{a}}_{\mathrm{obj}}||^{2} \rightarrow 0$). It is interesting to see that in all three scenarios it is always possible to make the object move. However, Scenario 2 (non-ideal passive wheels) has a limited region where the slippage ratio is high, compared to Scenario 3, where a whole region seems to give high values of slippage ratio. These regions in the input space are highlighted in Fig. 4b, c. Regions where the slippage of the snake robot are minimized tend to have higher slippage ratio.

### Case study 2: Snake robot with three joints

The proposed framework and metrics can be applied to a snake robot with any number of joints. In this section, a snake robot with three joints is studied. However, studying the three-dimensional input space could be cumbersome. Instead, the norms $||\vec {\varvec{\lambda }}||^{2}$, $||\vec {\varvec{a}}_{\mathrm{obj}}||^{2}$ and slippage ratio (30) are studied as a function of the polar coordinates of the COM of the snake robot $(|COM|,\angle COM)$ w.r.t. the contact point, as discussed in previous sections.

**Fig. 5:**
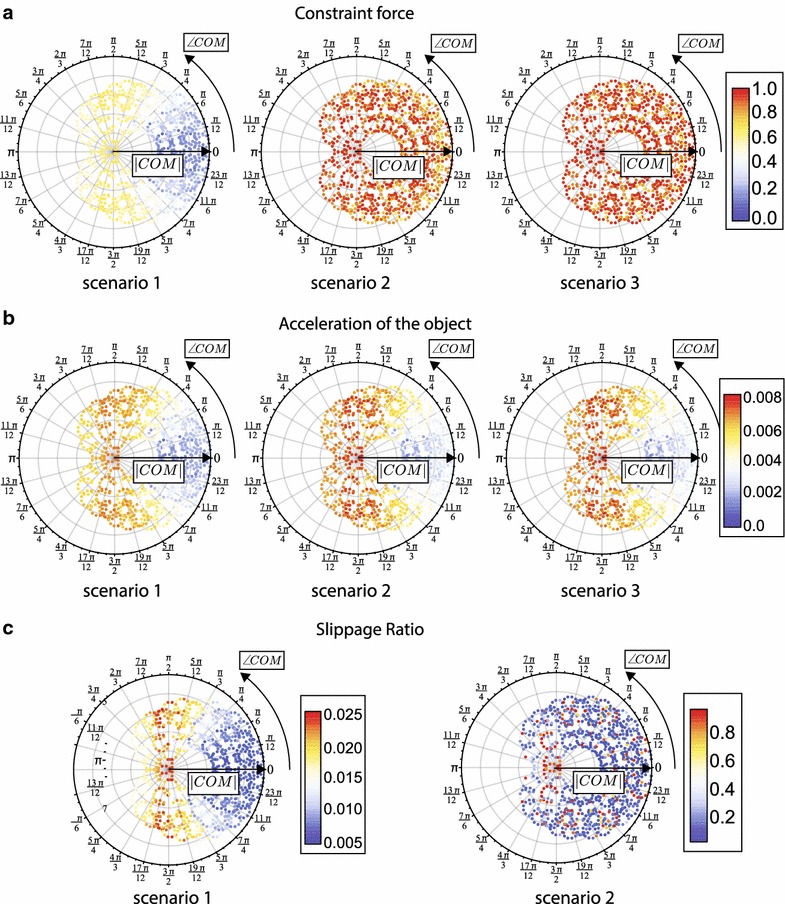
Norms studied over all configurations of the snake robot. **a** Constraint force (from left to right: scenarios 1, 2, and 3). **b** Acceleration of the object (from left to right: scenarios 1, 2, and 3). **c** Slippage ratio (from left to right: scenarios 1 and 2)

Figure 5a reports the result for the norm of constraint forces $||\vec {\varvec{\lambda }}||^{2}$. The polar plots show the results for scenario 1, 2, and 3, respectively. The higher the value, the bigger the constraint forces. It can be seen that in scenario 1 (negligible friction) there is a clear trend for configurations with the COM of the snake robot at angles $90^{\circ }$ and $-\,90^{\circ }$ to have a higher impact on the wrench applied to the object. Although scenarios 2 and 3 report a higher norm of the constraint forces, this is due to the addition of passive wheels. From this figure alone, it is not possible to ascertain the impact on the object.

Figure 5b reports the result for the norm of the object’s acceleration $||\vec {\varvec{a}}_{\mathrm{obj}}||^{2}$. The polar plots report the results for scenarios 1, 2, and 3, respectively. It can be seen that the addition of passive wheels (even ideal ones) have little impact on the acceleration of the object. However, the configuration of the snake robot, parametrized with the polar coordinates of its COM, has a clear and meaningful impact on the acceleration of the object.

Although basic intuition would tell that the addition of constraints (i.e., passive wheels) should have an impact on the force applied to the object (through $\vec {\varvec{\lambda }}$), and consequently on its acceleration $\vec {\varvec{a}}_{\mathrm{obj}}$, this study shows that is not the case (at least, not that simply).

An important addition of this paper w.r.t. [15, 24] is the study of the slippage ratio sr. By studying the relationship between motions of both systems (snake robot and object), we can understand how the additional constraints have an impact on the system. A snake robot without passive wheels will slip as it pushes the object. Therefore, minimizing this motion while keeping a steady force on the object (and therefore producing an acceleration) is desirable. Figure 5c shows the result of the slippage ratio (30). It can be seen that in Scenario 1 (without passive wheels) the same trend as with the object’s acceleration appears. However, passive wheels (even non-ideal ones) have a big impact on the slippage ratio (take notice of the change of scale).

**Fig. 6:**
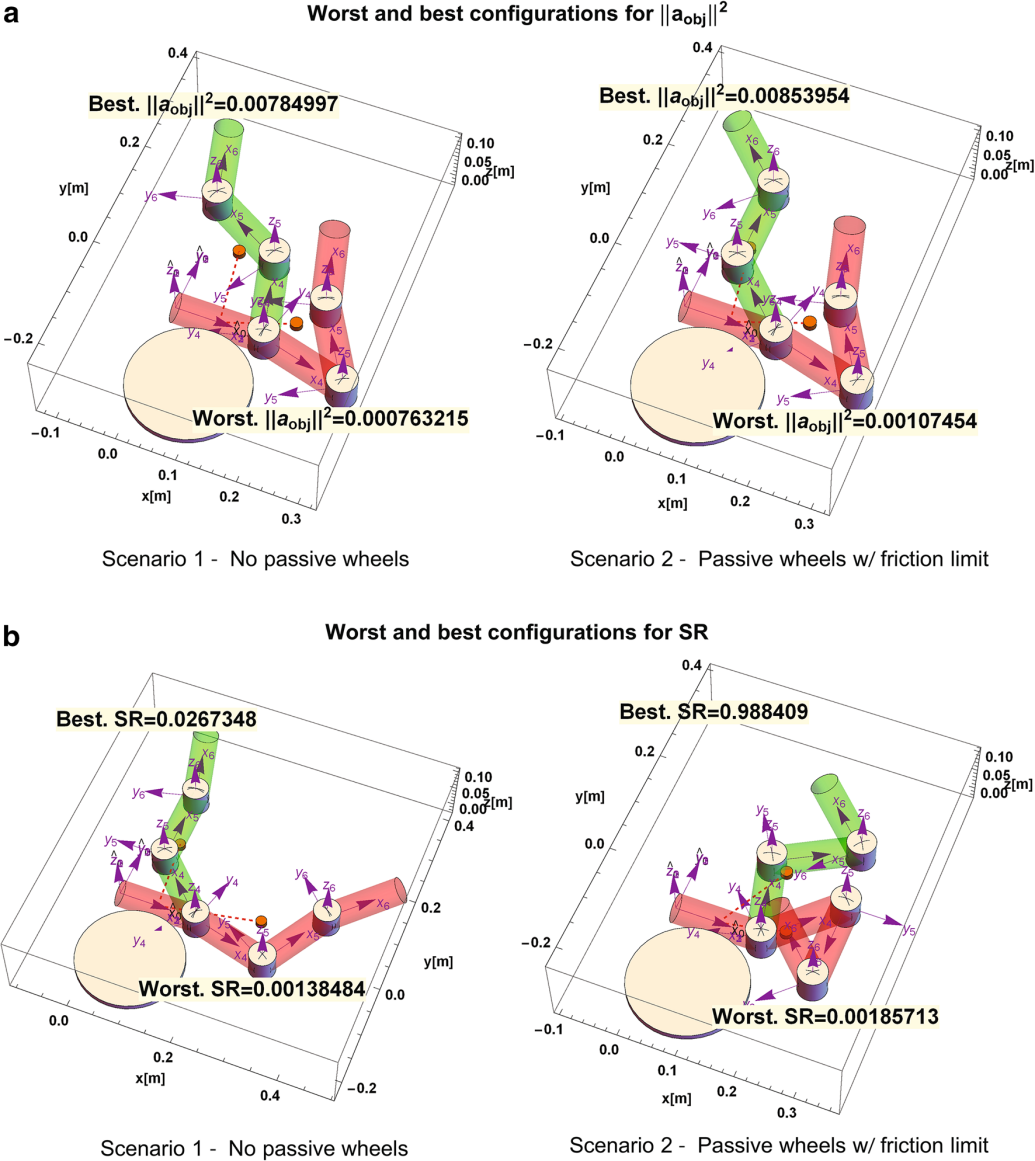
Representative configurations chosen among the best and worst configurations of the snake robot. **a** Acceleration of the object. **b** Slippage ratio

To show more clearly the impact of the configuration of the snake robot on the acceleration and slippage of the system, Fig. 6 shows the best and worst configurations for the acceleration of the object Fig. 6a and for slippage ratio Fig. 6b. The results are summarized in Table 2. It can be seen that passive wheels (Scenario 2 and 3) have little impact on the acceleration, but a significant one on decreasing the slippage of the snake robot ($\hbox {sr} \rightarrow 1$).

**Table 2:** Results of the norms

Concept	Scenario 1	Scenario 2	Scenario 3
Worst $||\vec {\varvec{a}}_{\mathrm{obj}}||^{2}$	0.000763215	0.00107454	0.00107454
Best $||\vec {\varvec{a}}_{\mathrm{obj}}||^{2}$	0.00785302	0.00861168	0.00861168
Worst sr	0.00138484	0.00185713	0.00185098
Best sr	0.0267348	0.988638	0.988643

## Discussion and future applications

Several assumptions have been made in this line of research, especially the number of contacts considered between objects, and their rigidity. This is because we are interested in giving a solution that is mathematically rigorous while guaranteeing uniqueness of solution. To include more contact points means to loose this in favor of robustness. For example, a penalty method (aka. virtual springs) or barrier functions may be considered, which is common for whole-arm body manipulation (WAM) tasks. Although our assumptions are restrictive, it allows us to give a solid foundation for the research. Other models or considerations can be used for more realistic scenarios, but the rigid-body assumption used here allows to have a clear basis for comparison. Considering the gaps in knowledge regarding snake robots (as highlighted in “Background” section), we consider the model and results presented to be useful for moving research forward.

The holonomic constraints (e.g., constraints due to joints of the snake robot) are already encoded in the kinematic model of the snake robot presented in “Mathematical background” section. These holonomic constraints are described by the use of the robot’s Geometric Jacobians. Further distinction between holonomic and non-holonomic constraints is not necessary, as both can be expressed in the same unified manner (13), as mentioned in [21, 33].

The metrics and general framework presented can be used to analyze more complex tasks involving snake robots (or similar robotic systems) interacting with an object or the environment. This gives an opportunity to study both snake robots and robotic arms in the same framework, since the analysis is similar to the often-used force/manipulability ellipsoids used to study robotic arms or hands [34].

However, the metrics presented in previous research do not consider the motion of the robot itself, since a fixed-base was always assumed. The framework presented in this paper can be applied to other mobile systems in a more complete manner than reported in the literature [28, 29]. More specifically, the analysis presented extends the concept of force or manipulability ellipsoids [26, 34], from the case of a fixed-base robot with end-effector, to a mobile robot without an end-effector. For a given task and configuration, an analysis can be carried out to find the optimal input (vector of joint torques) to minimize or maximize slippage of the system.

A few conclusions can be drawn from analyzing norms (26) and (27) on the input space (c.f. Fig. 4). In all scenarios, $\tau _{2}$ which is the joint furthest away from the object has almost no effect on the acceleration of the object. However, the addition of passive wheels helps to *anchor* the snake robot and couples the effect of $\tau _{2}$ on the system.

## Conclusion

In this paper, a modeling and analysis framework for snake robots in contact with an external body has been presented. Results show that the addition of passive wheels has little effect on the wrench applied to the object and therefore, little change in its acceleration. However, the passive wheels do have an effect on the motion of the robot itself. In other words, under certain conditions the slippage of the robot can be minimized while pushing the object. This could be beneficial for pushing or manipulation tasks. To the best of our knowledge, this problem has not been fully studied with snake robots.
